# Supplementary material for: The Experimental Infection of Goats with Small Ruminant Morbillivirus Originated from Barbary Sheep
Source: Pathogens. 2022 Aug 30;11(9):991. doi: 10.3390/pathogens11090991 (PMC9502496; doi:10.3390/pathogens11090991)

**Figure S1-A.** ID rapid PPR antigen dipstick field test results of nasal swab. Negative (-) control band visible test band not -, questionable ((+)) control band visible and barely visible test band, low positive (+) test band visible but not strong colour as control band, moderate positive (++) test band same visible colour as control band, high positive (+++) test band stronger colour than control band.

Nasal swab 0 dpi

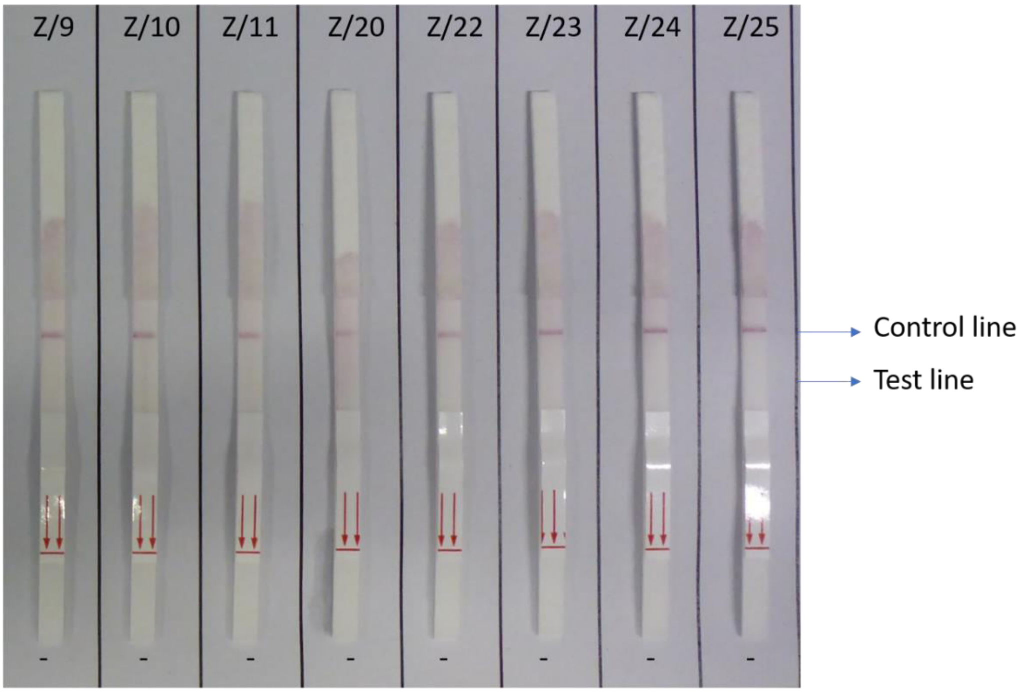


Nasal swab 3 dpi

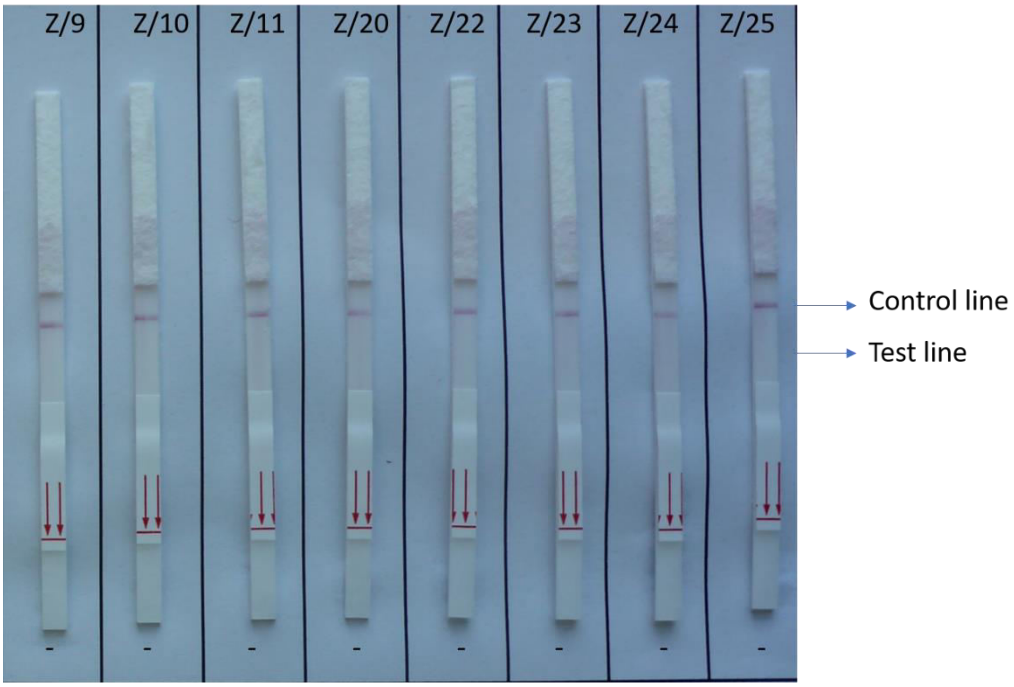

Nasal swab 5 dpi

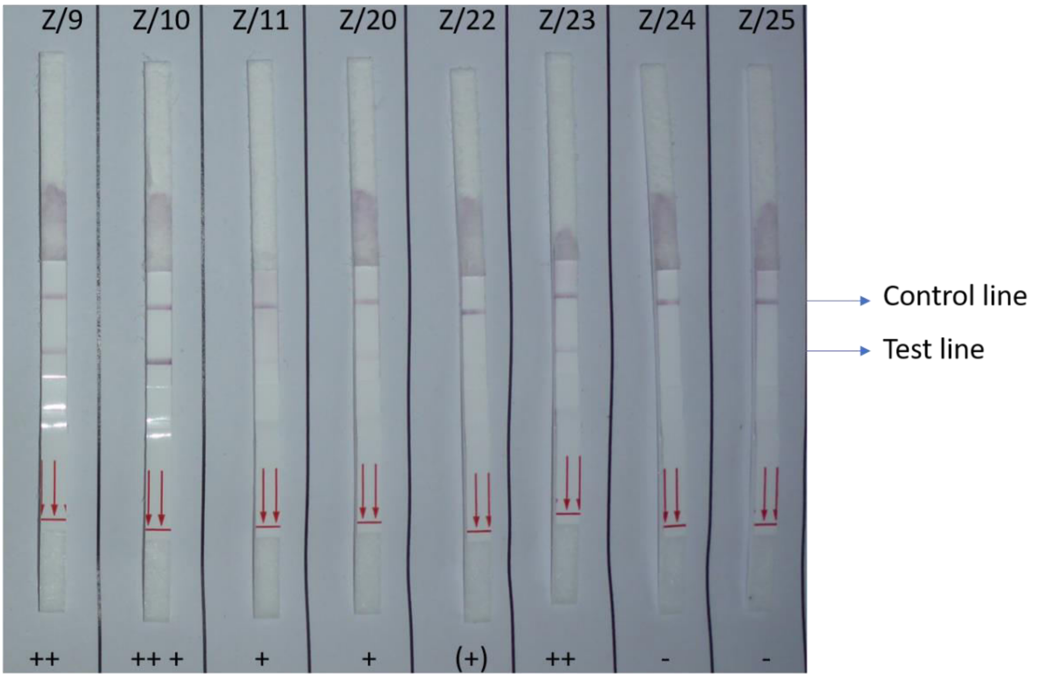


Nasal swab 7 dpi

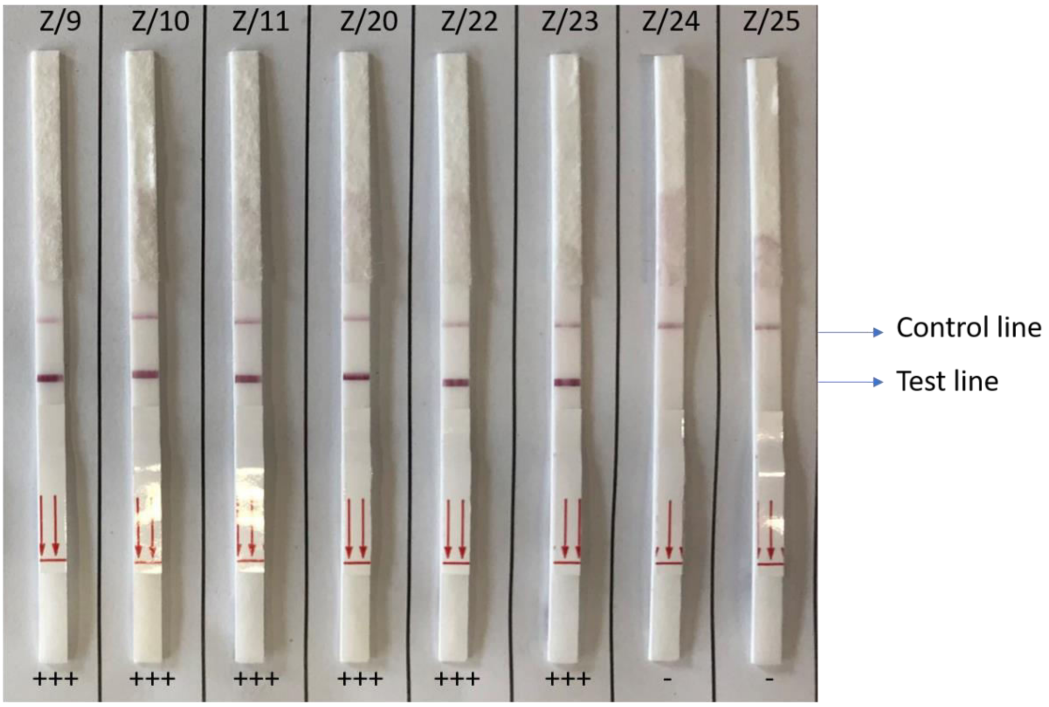

Nasal swab 10 dpi

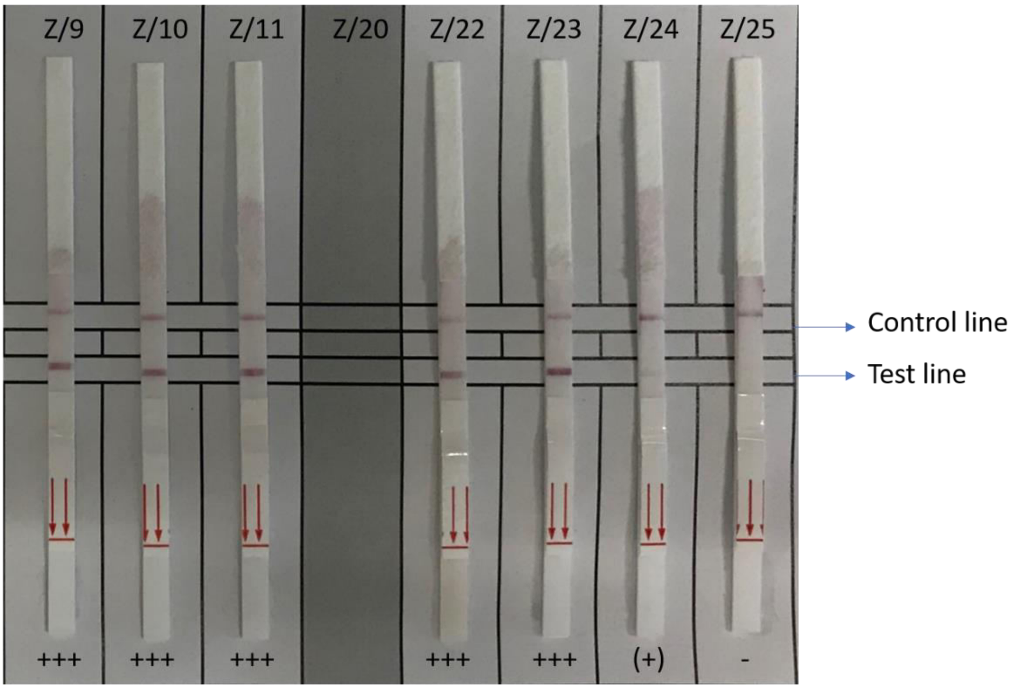


Nasal swab 12 dpi

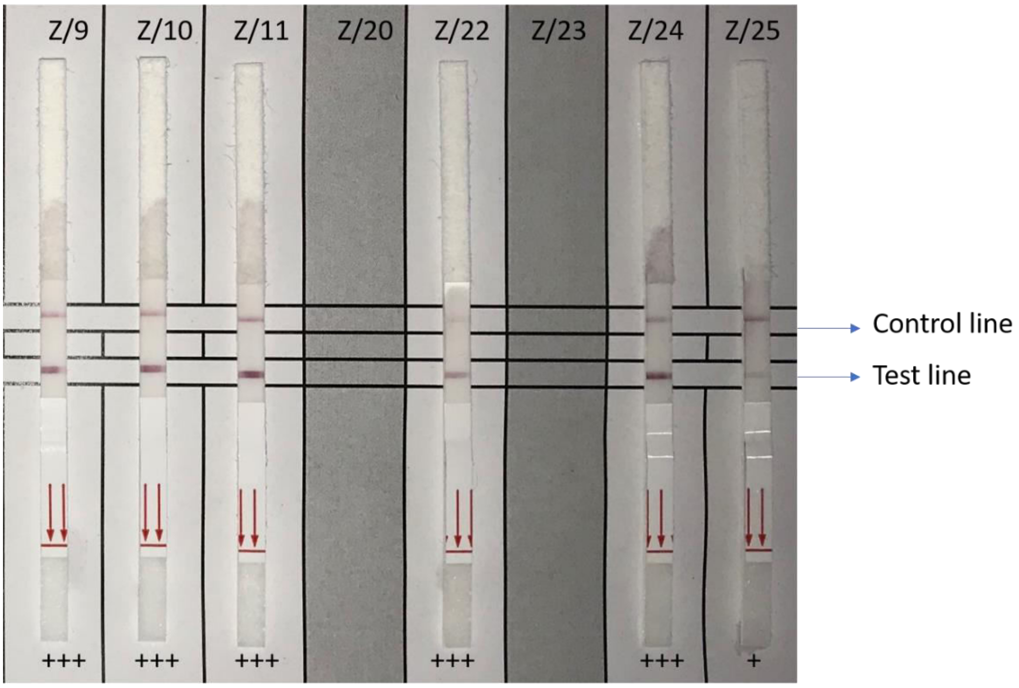

Nasal swab 14 dpi

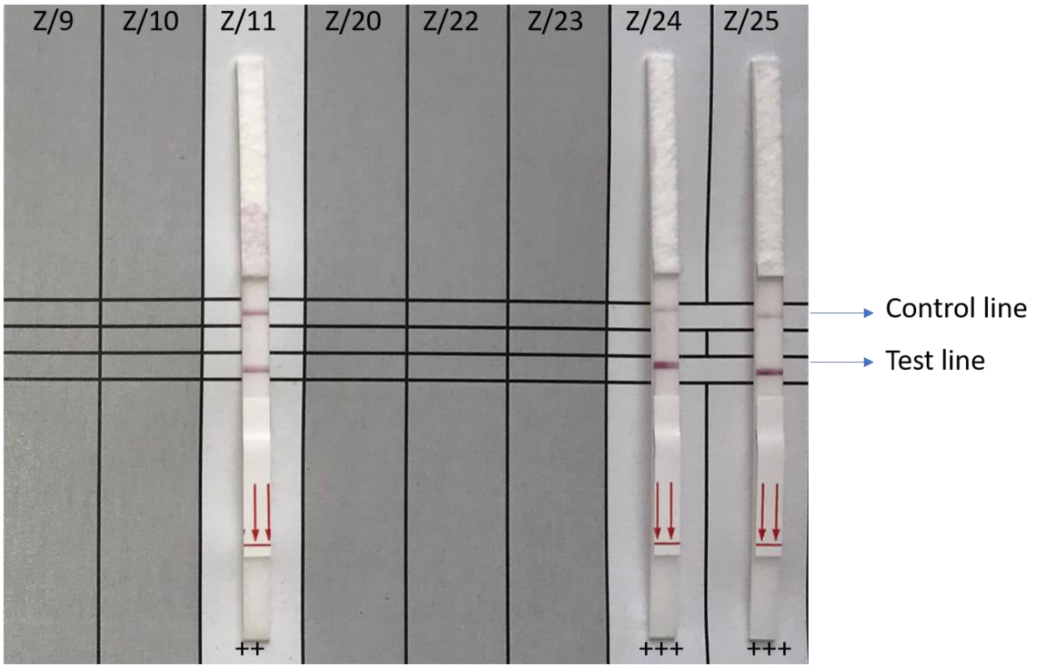


Nasal swab 17 dpi

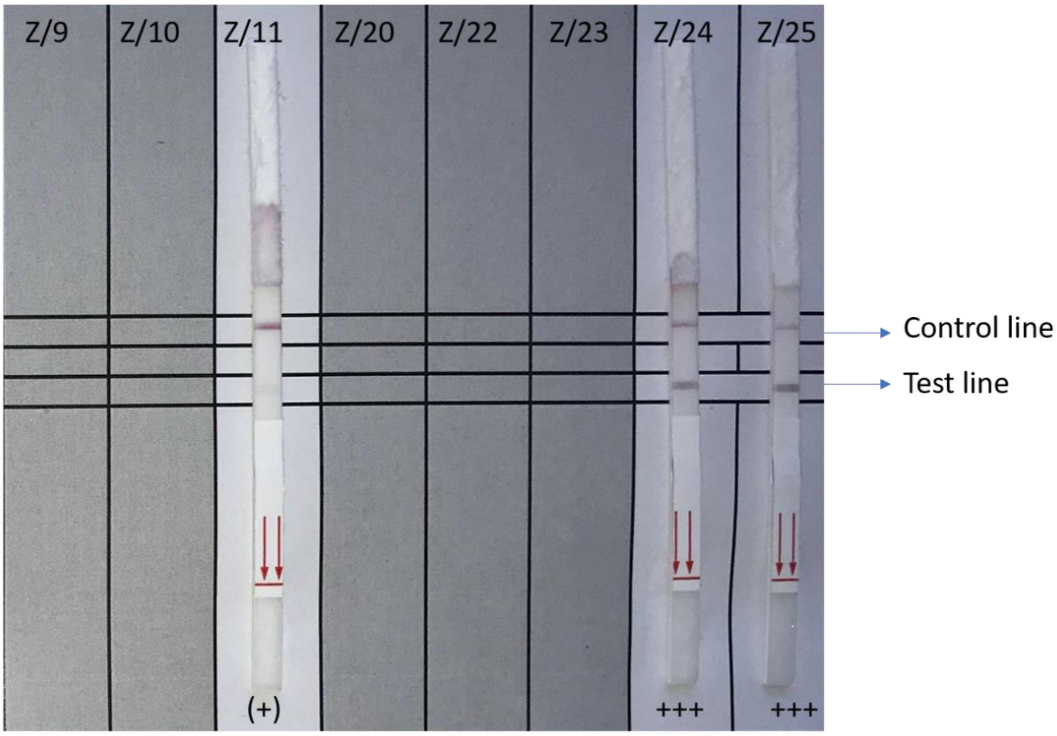

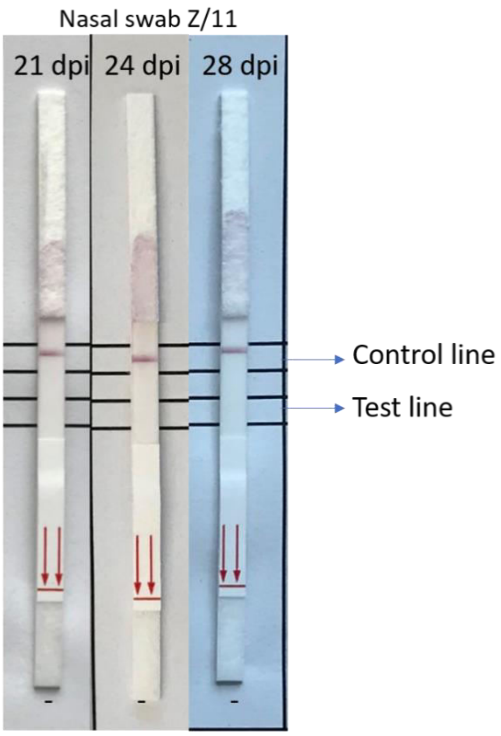
Nasal swab Z/11
21 dpi
24 dpi
28 dpi
Control line
Test line
-
-
-

**Figure S1-B.** ID rapid PPR antigen dipstick field test results of conjunctival swab. Negative (-) control band visible test band not -, questionable ((+)) control band visible and barely visible test band, low positive (+) test band visible but not strong colour as control band, moderate positive (++) test band same visible colour as control band, high positive (+++) test band stronger colour than control band.

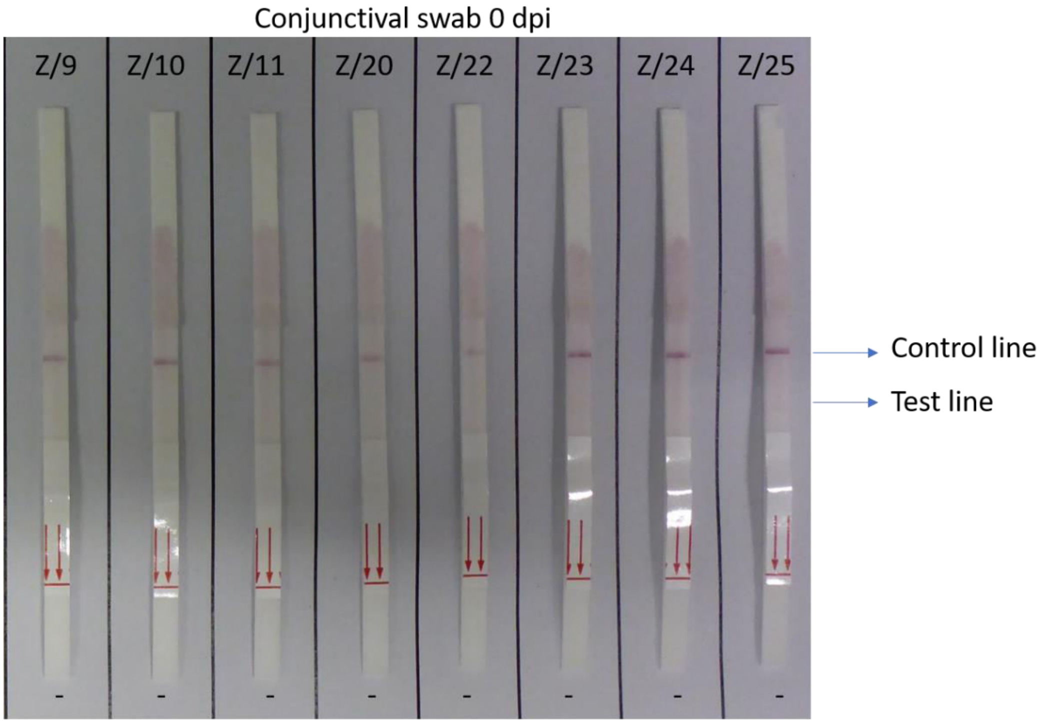


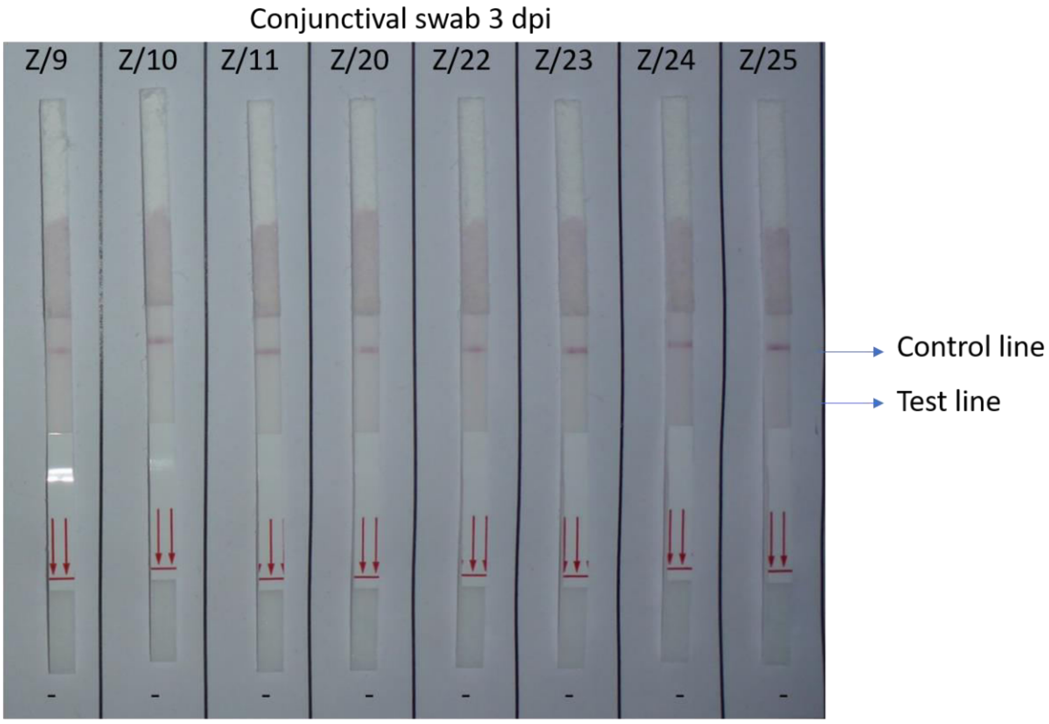

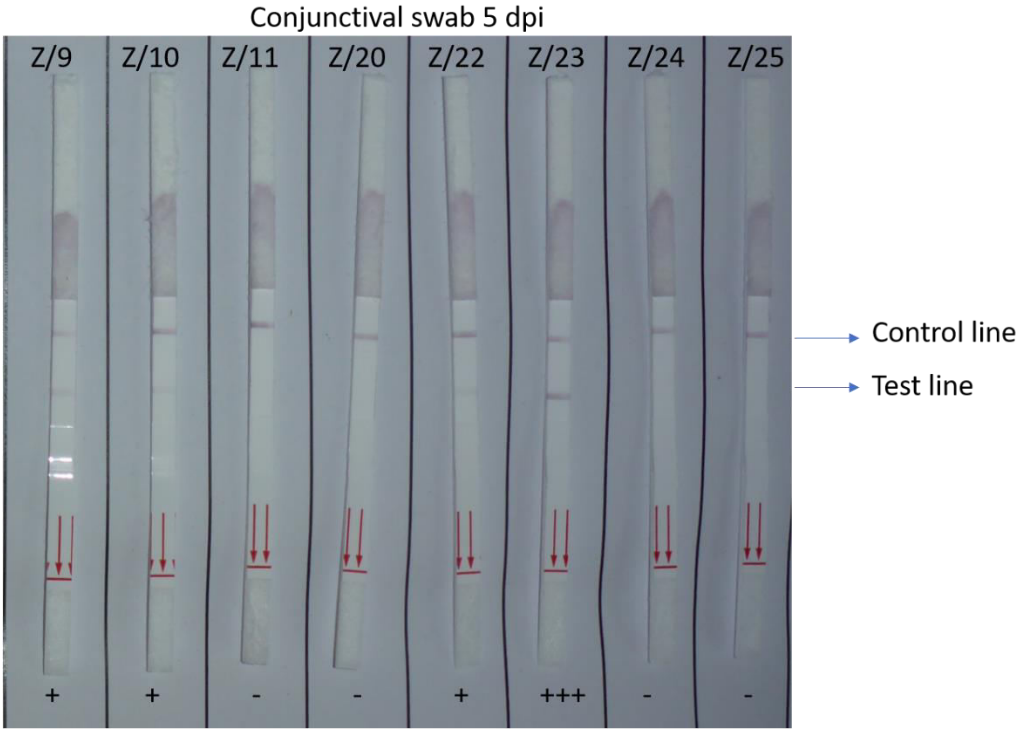
Conjunctival swab 5 dpi
Z/9
Z/10
Z/11
Z/20
Z/22
Z/23
Z/24
Z/25
+
+
-
-
+
+++
-
-
Control line
Test line

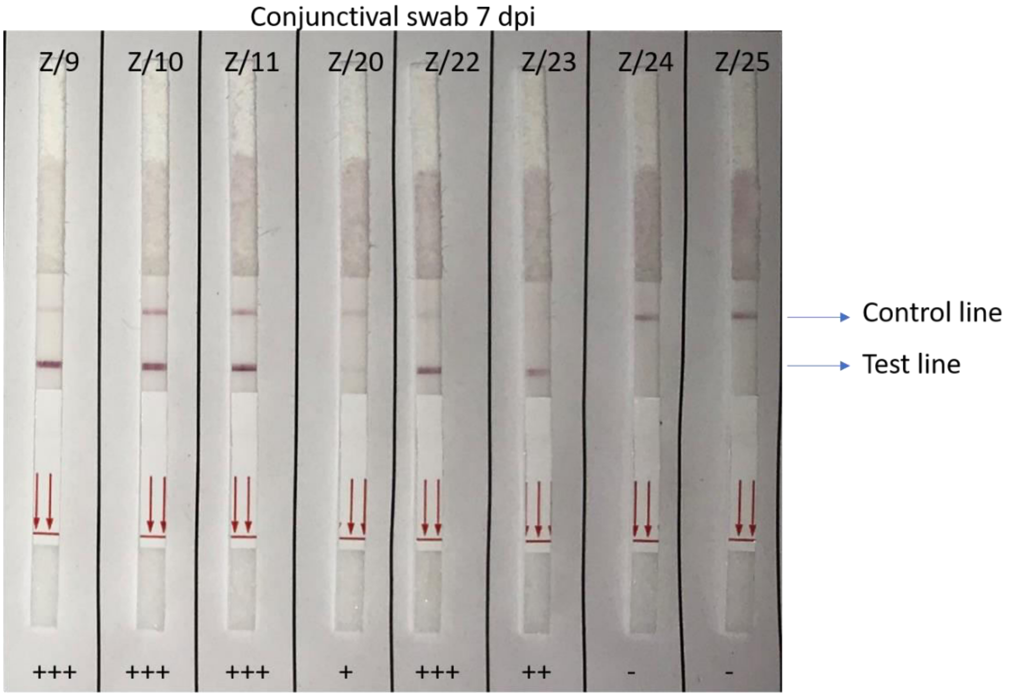
Conjunctival swab 7 dpi
Z/9
Z/10
Z/11
Z/20
Z/22
Z/23
Z/24
Z/25
+++
+++
+++
+
+++
++
-
-
Control line
Test line

Conjunctival swab 10 dpi

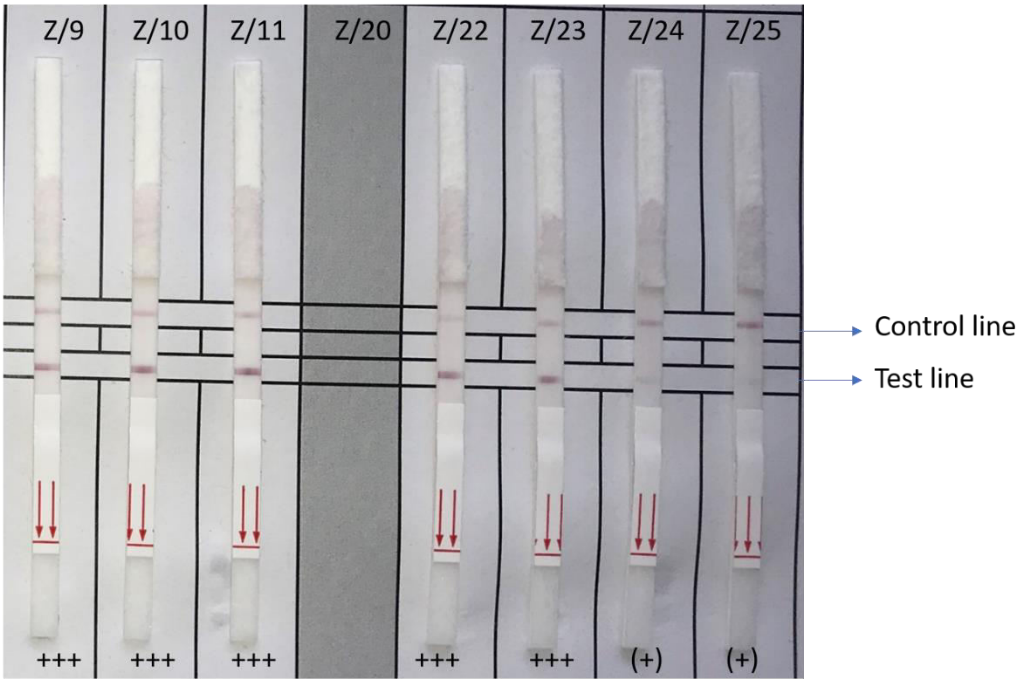


Conjunctival swab 12 dpi

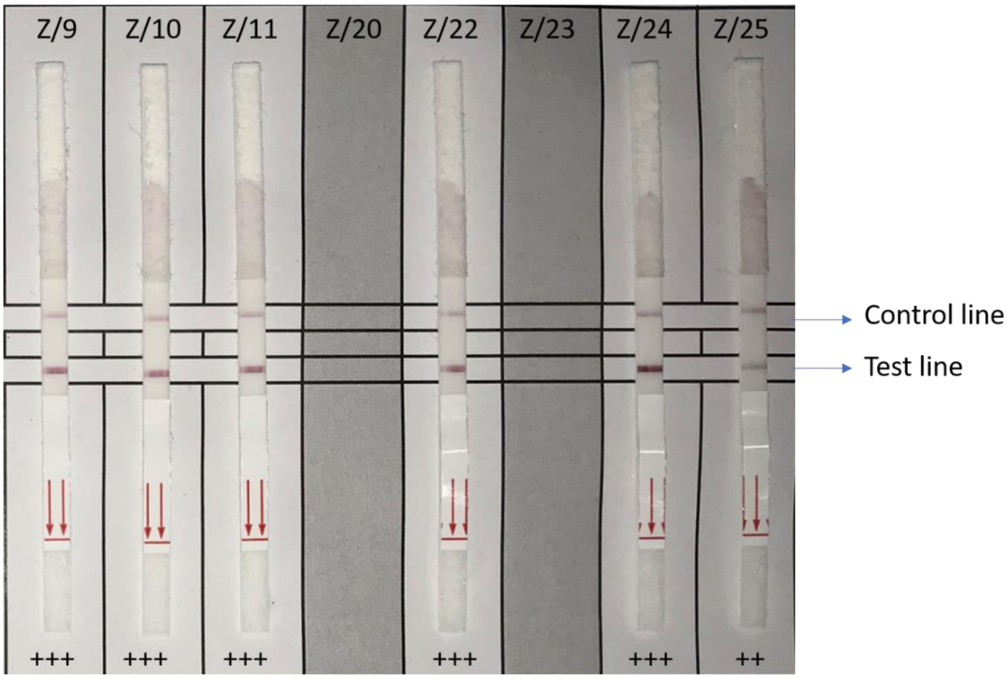

Conjunctival swab 14 dpi

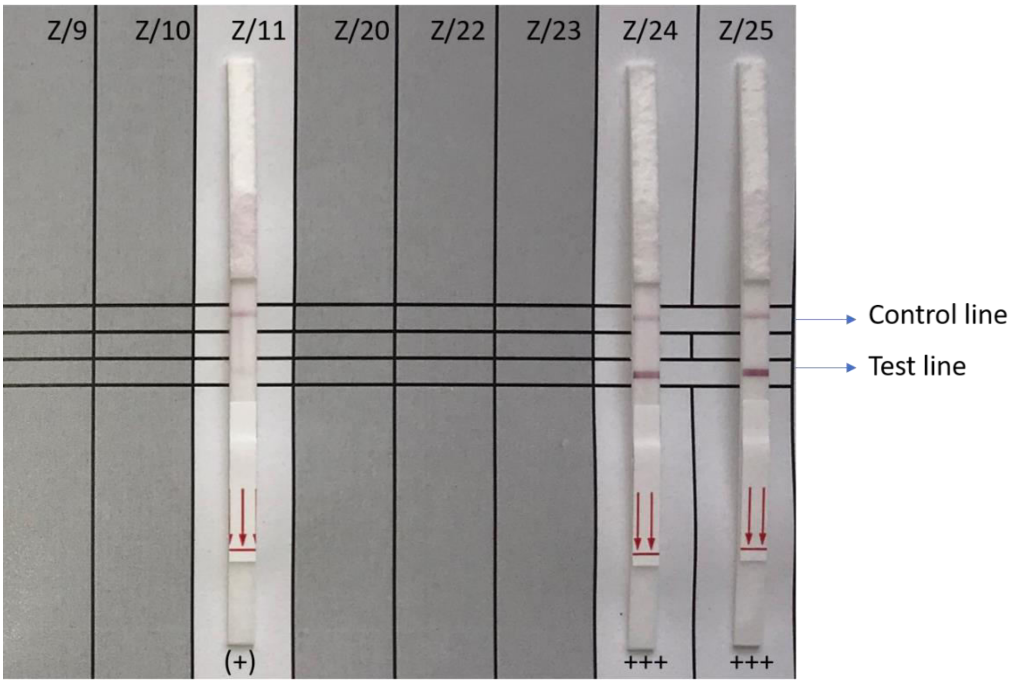


Conjunctival swab 17 dpi

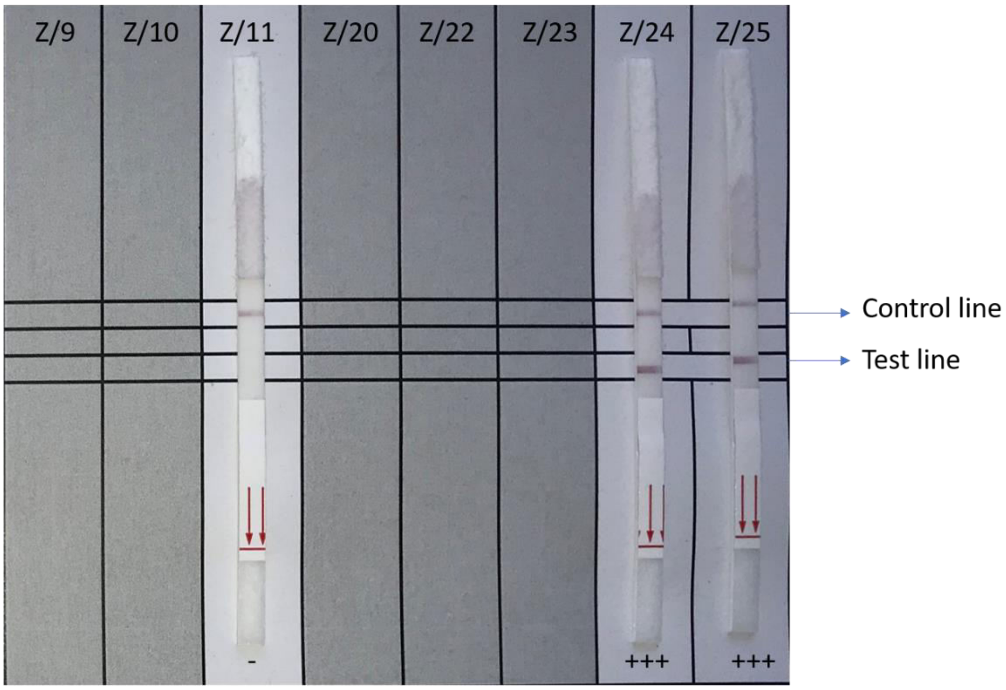

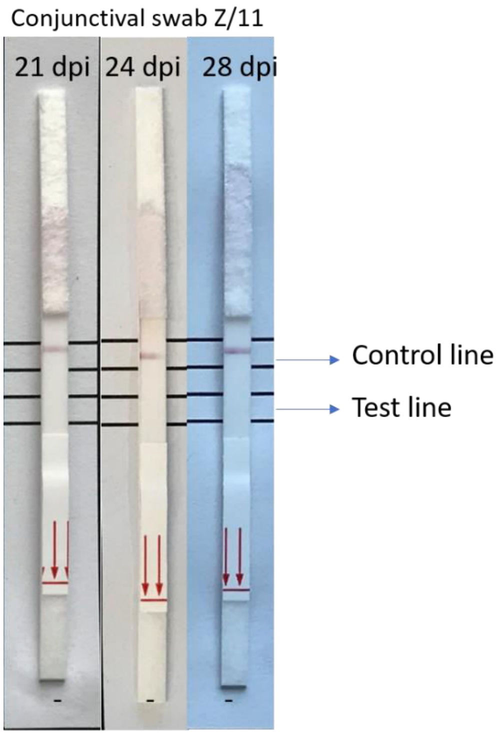
Conjunctival swab Z/11
21 dpi
24 dpi
28 dpi
Control line
Test line

**Figure S1-C.** ID rapid PPR antigen dipstick field test results of live and antibody neutralized SRMV cell culture isolate. Negative (-) control band visible test band not -, questionable ((+)) control band visible and barely visible test band, low positive (+) test band visible but not strong colour as control band, moderate positive (++) test band same visible colour as control band, high positive (+++) test band stronger colour than control band.

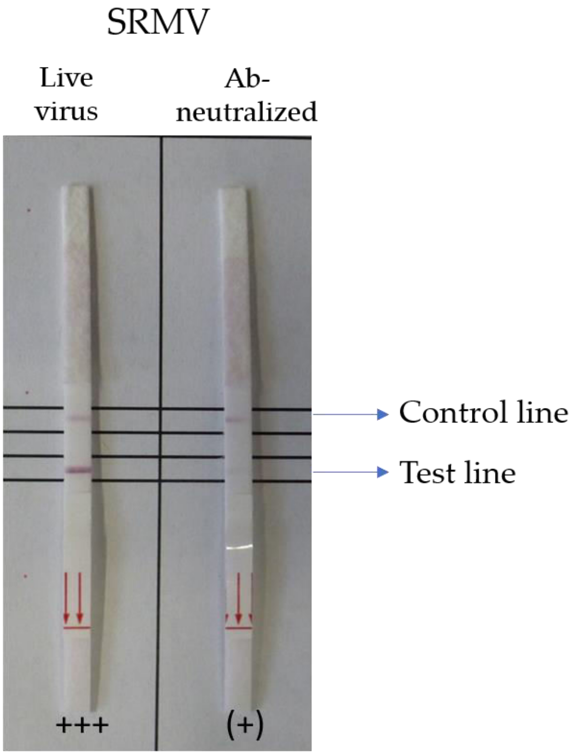

Supplement: Supplementary file 1 [file pathogens-11-00991-s001.zip › pathogens-1872500-supplementary.pdf]
